# Continous, non-invasive monitoring of oxygen consumption in a parallelized microfluidic *in vitro* system provides novel insight into the response to nutrients and drugs of primary human hepatocytes

**DOI:** 10.17179/excli2021-4351

**Published:** 2022-01-07

**Authors:** Marius Busche, Dominik Rabl, Jan Fischer, Christian Schmees, Torsten Mayr, Rolf Gebhardt, Martin Stelzle

**Affiliations:** 1 NMI Natural and Medical Sciences Institute at the University of Tübingen, Reutlingen, Germany; 2 Institute of Analytical Chemistry and Food Chemistry, Graz University of Technology, Graz, Austria; 3 PyroScience AT GmbH, Aachen, Germany; 4 Rudolf-Schönheimer-Institute of Biochemistry, Leipzig University, Leipzig, Germany; 5 InViSys-Tübingen GbR, Leipzig, Germany

**Keywords:** liver, organ-on-chip, perfusion, oxygen, sensors, in vitro, model, toxicity, metabolism

## Abstract

Oxygen plays a fundamental role in cellular energy metabolism, differentiation and cell biology in general. Consequently, *in vitro* oxygen sensing can be used to assess cell vitality and detect specific mechanisms of toxicity. In 2D *in vitro* models currently used, the oxygen supply provided by diffusion is generally too low, especially for cells having a high oxygen demand. In organ-on-chip systems, a more physiologic oxygen supply can be generated by establishing unidirectional perfusion. We established oxygen sensors *in* an easy-to-use and parallelized organ-on-chip system. We demonstrated the applicability of this system by analyzing the influence of fructose (40 mM, 80 mM), ammonium chloride (100 mM) and Na-diclofenac (50 µM, 150 µM, 450 µM, 1500 µM) on primary human hepatocytes (PHH). Fructose treatment for two hours showed an immediate drop of oxygen consumption (OC) with subsequent increase to nearly initial levels. Treatment with 80 mM glucose, 20 mM lactate or 20 mM glycerol did not result in any changes in OC which demonstrates a specific effect of fructose. Application of ammonium chloride for two hours did not show any immediate effects on OC, but qualitatively changed the cellular response to FCCP treatment. Na-diclofenac treatment for 24 hours led to a decrease of the maximal respiration and reserve capacity. We also demonstrated the stability of our system by repeatedly treating cells with 40 mM fructose, which led to similar cell responses on the same day as well as on subsequent days. In conclusion, our system enables in depth analysis of cellular respiration after substrate treatment in an unidirectional perfused organ-on-chip system.

## Introduction

Hepatocytes exhibit a high demand for oxygen despite their prevalent supply by venous blood from the gut. In static cell cultures, however, oxygen supply provided by diffusion is generally too low (Scheidecker et al., 2020[43]; Stevens, 1965[45]) leading to suboptimal metabolic rates. On the other hand, cultivation at low oxygen (5 %) concentration can reduce dedifferentiation of primary mouse hepatocytes in vitro when compared to cultures maintained at 21 % oxygen tension (Guo et al., 2017[20]). Hyperoxia, however, was shown to support the differentiation status of hepatocyte cell lines such as HepaRG and C3A as evident from measurements of CYP activity and protein expression (van Wenum et al., 2018[50]). Ast et al. expect culturing cells at physiological oxygen concentration to yield a more robust cellular model and improve *in vitro in vivo* correlation (Ast and Mootha, 2019[3]). 

Thus, the integration of oxygen sensing in microphysiological systems might serve in generating a more physiological microenvironment and provide insight into oxygen-dependent cell function. As cellular respiration also is an important marker for cell viability, oxygen sensing can be used to assess the influence of drugs or toxic agents on mitochondrial function (Hynes et al., 2003[22]; O'Riordan et al., 2000[37]). Drug-induced mitochondrial dysfunction is a major cause for liver pathophysiology (Degli Esposti et al., 2012) and can be determined by measuring respiration (Hynes et al., 2006[23]).

Two commercialized benchmark systems to measure respiration rates in *in vitro* systems are the MitoXpress platform and the seahorse XF analyser (Ferrick et al., 2008[18]; Hynes et al., 2009[24]), both from Agilent (Santa Clara, CA, United States). The MitoXpress platform uses standard 96-wellplates with dispensed oxygen sensitive probes (Hynes et al., 2009[24]). The wells are sealed with oil and can be read out by a fluorescence plate reader. This system does not easily enable consecutive treatments and lipophilic substances in the medium can be lost to the oil phase. However, the seahorse XF platform uses customized 96-wellplates which can be sealed by a plunger and enables up to 4 automated consecutive substance injections during continuous measurement (Ferrick et al., 2008[18]). But still, this system does not implement perfusion and continuous medium exchange. This hinders the building of more *in vivo*-like biochemical microenvironments and gradients.

Oxygen supply to cell cultures can be controlled and monitored by employing microfluidic cell culture devices (for a review, see Low et al., 2020[29]). By using materials that are impermeable to oxygen, its supply to the cells by diffusion through the walls of the device can be prevented (Zirath et al., 2018[56]). Thus, oxygen is provided solely through medium perfusion and can be precisely adjusted by controlling the perfusion rate. Under these conditions, the oxygen concentration inside the cell chamber only depends on the initial oxygen concentration at the inlet of the chamber, the oxygen consumption (OC) of the cells and the flow rate. The medium supplied through medium reservoirs will equilibrate its oxygen concentration with the incubator atmosphere. Thus, by measuring the oxygen concentration at the inlet and the outlet of the cell culture chamber, the OC of the cultured cells can be precisely determined. OC has already been measured in microfluidic cell culture devices demonstrating expected cell responses on e.g. cytochalasin B, chloracetaldehyde (Brischwein et al., 2003[7]), stausporine (Rennert et al., 2015[42]) and FCCP (Müller et al., 2021[33]). These systems mostly require complex tubing which renders handling tedious. To enable a broad adoption of microfluidic systems with integrated oxygen sensing methods, convenient systems are needed. These systems should keep up unidirectional perfusion during the measurement to preserve the equilibrium of the cellular microenvironment.

Currently, the readout systems used for oxygen concentration in microfluidic cell culture devices employ either electrochemical or optical oxygen sensors. Electrochemical sensors benefit from their straightforward integration of miniaturized electrodes in microfluidic systems by processes established in microelectronics (Brischwein et al., 2003[7]). Newer approaches make use of ink-jet printing techniques (Moya et al., 2018[32]). The sensing element of optical oxygen sensors is composed of a phosphorescent dye embedded in a host polymer (for review see Papkovsky and Dmitriev, 2013[38]). Oxygen quenches the phosphorescence of the dye causing a change in the intensity and lifetime of the excited state. In contrast to electrochemical sensors, optical oxygen sensors do not consume the analyte during the measurement and do not require an additional reference element. Optical sensing elements can be read-out contactless via optical fibers from the outside through the wall of the microfluidic cell culture chamber. For detailed reviews of oxygen sensors and their integration in cell culture devices, the reader is referred to the following authors (Gruber et al., 2017[19]; Kieninger et al., 2018[26]; Oomen et al., 2016[36]; Ungerböck and Mayr, 2018[47]).

Recently, we have developed an easy-to-use organ-on-chip system in the SBS-standard well plate format with 24 independent cell culture chambers for permanent perfusion with culture medium - the HepaChip-MP (Busche et al., 2020[8]). In the present work, we demonstrate the non-invasive, continuous and parallelized measurement of the OC of primary human hepatocytes (PHH) after being challenged by diverse metabolic substrates and drugs. This was enabled by integrating oxygen sensing elements at the inlet and the outlet of each chamber, respectively. Unidirectional perfusion of medium results in constant substance supply in the cellular microenvironment. We demonstrate the applicability of our system by analyzing the response of cellular OC to the application of glucose and fructose as well as ammonium chloride and the drug diclofenac. PHH showed an immediate drop of OC after fructose treatment. This effect was not observed after treatment with glucose. Through in depth analysis of mitochondrial respiration after substance treatment, our system also enables insights into the mode of action of ammonium chloride and diclofenac toxicity. 

## Material and Methods

### Integration of sensors in HepaChip-MP

Phosphorescent sensor spots were prepared by applying a sensor formulation with a microdispenser MDS3200+ from VERMES Microdispensing GmbH, equipped with a 70 µm nozzle and a tungsten tappet with a tip diameter of 0.7 mm onto polymeric substrate. The microdispenser was mounted on a custom-made CNC platform, which was controlled via Linux CNC and allowed exact positioning of the sensor spots. The sensor formulation consists of 1.12 mg of platinum(II)meso-tetra(4-fluorophenyl) tetrabenzoporphyrin (PtTPTBPF) and 100 mg polystyrene (PS, 26000 g/mol) dissolved in 900 mg in toluol. 

### Priming of chip and seeding of cells

All steps, which are not stated as “manually” were performed with a pipetting robot (CyBio FeliX, Analytik Jena, Jena, Germany) modified by Ionovation (Bissendorf, Germany). The priming and handling of the HepaChip-MP (Microfluidic ChipShop, Jena, Germany) has already been described in detail in Busche et al. (2020[8]). Briefly, the chambers were filled with 1 mg/ml Pluronic solution in HEPES buffer (pH 7.4). Afterwards, the chip was flushed with Collagen-Pluronic solution (100 µg/ml Collagen, 1 mg/ml Pluronic) in HEPES buffer (pH 4). The inlet tanks were filled manually with 500 µl Collagen-Pluronic solution and the chip was incubated overnight at room temperature to ensure coating of the assembly ridges. On the next day, the Collagen-Pluronic solution was manually removed from the inlet and outlet tanks. Dielectrophoresis (DEP) medium was flushed through the chambers and subsequently, the cells were assembled. Cells were manually prepared as described in 'Cell preparation for seeding inside the HepaChip-MP' and assembled by dielectrophoresis (V_pp_ = 80 V, f = 360 kHz). Afterwards, cell culture medium was flushed through the chambers and the inlet tanks were manually filled with 500 µl cell culture medium. After 6 hours, perfused medium and cells were removed and the inlet tank filled up to approximately 900 µl. Cells were used for experiments on days 2 and 3 of culture.

### Cell preparation for seeding inside the HepaChip-MP

Cryopreserved primary human hepatocytes were purchased from Cytes Biotechnologies (Barcelona, Spain) (Donors BHuf16068 and BHuf16029). For thawing, cryopreserved cells were kept in a water bath at 37 °C until only a small frozen area was left. Then, cells were transferred to a 15 ml centrifugation tube filled with 7 ml preheated cell culture medium. Cell culture medium was Williams E with glutamax supplemented with the following: 10 % FBS, 15 mM HEPES, 1 mM sodium pyruvate, 1 % MEM non-essential amino acids, 1 µg/ml dexamethasone, 1 µg/ml insulin, 0.55 g/ml human transferrin, 0.5 ng/ ml sodium selene, 53.5 mg/ml linoleic acid, 100 µg/ml Primocin. The transferred cells were centrifuged at 50 x g at room temperature for 5 minutes and the supernatant was removed. Afterwards, the cell pellet was resuspended in cell culture medium. Cell number and viability were determined by trypan blue exclusion test. 4 Mio hepatocytes were transferred to another 15 ml centrifugation tube and DEP medium was added to a final volume of 10 ml. Afterwards, cells were centrifuged at 50 x g at room temperature for 5 minutes. The supernatant was removed and the cell pellet resuspended in 2 ml DEP medium. This cell suspension was used to assemble the cells in the HepaChip-MP.

### Routine culture

The perfusion in the HepaChip-MP is initiated by gravity. By filling the inlet tank to a higher level than the outlet tank, the medium perfuses from the inlet tank through the cell culture chamber to the outlet tank (Supplementary Figure 1). Consequently, the height difference between medium level in inlet and outlet had to be periodically readjusted during routine culture. Between readjustments, the flow velocity constantly decreases (Supplementary Figure 1). We readjusted the height difference between medium levels in the inlet and outlet tanks at least every 14 hours to ensure flow velocities higher than ~10 µl/h. Medium that is used with the HepaChip-MP in the incubator should always be equilibrated to the temperature and the atmosphere of the incubator to prevent formation of gas bubbles in the microfluidic device. 

### Oxygen measurements

#### Calibration

Calibration data for the oxygen sensor spot were similar to those reported in Ehgarnter et al. (2016[16]). The oxygen sensors were calibrated by a two‐point calibration. The microfluidic chamber was flushed with gasous N_2_ to determine the phase shift (dphi) at deoxygenated conditions. To define the phase shift (dphi) at air saturation, the last value after treatment with rotenone+antimycin A was employed. 

#### Measurement

Experiments were carried out on day two and three after cell assembly. At the beginning of the experiment, the culture medium was removed from the inlet and outlet tanks. Then, the inlet tanks were filled with 900 µl of cell culture medium and the chip was placed on the readout platform in the incubator. Afterwards, the measurement was started.

For substance treatments, the chip was incubated until a baseline was reached before the substance of interest was added. To add the substance, the medium from inlet and outlet tanks was removed and 900 µl of medium supplemented with the substance of interest was added. For successive treatments, the medium with the first substance was removed and the medium with the new substance (or medium only) was filled in the inlet tanks. Substances used, their concentration and incubation time are presented in Table 1(Tab. 1) (References in Table 1: Hengstler et al., 2020[21]; Aduen et al., 1994[1]; Nelson et al., 2011[34]; Divakaruni et al., 2014[14]; Cooper et al., 1989[9]; Davies and Anderson, 1997[12]).

The oxygen measurements were performed with a customized multi-channel device obtained from PyroScience GmbH (Aachen, Germany). The miniaturized instrument provides 48 optical oxygen channels in a robust aluminium housing with a dimension of ca. 155 x 124 x 30 mm. The device features fiber-optic ST-receptacles that accept 1 mm-core diameter polymer optical fibers for readout of the luminescent sensor spots. The technology is based on the proprietary “red-flash”-Technology used in other PyroScience devices. Each channel consists of a red excitation LED (λ_peak_=624 nm), a short-pass excitation filter (λ_cut_ = 690 nm), and focusing optics to couple the excitation light into the optical fiber. The luminescent light from the sensor spot travels through the same fiber back to the instrument. A long-pass emission filter (λ_cut_ = 720 nm) blocks all reflected or back-scattered excitation light that reaches the detector directly from the LED. A photodiode captures the luminescence of the sensor spot. The photodiode current is amplified, digitized by an A/D converter, and analyzed by the microcontroller of the device. The LED current is modulated with sinusoidal light, and therefore the emitted light is modulated likewise. Due to the luminescence lifetime of the sensor material, there is a phase shift between excitation and emission light. The luminescence lifetime and therefore the phase shift is quantitatively coupled to the oxygen partial pressure of the sensor by a proprietary, modified version of the Stern-Volmer equation. The instrument reads out the 48 channels sequentially. Depending on the measurement time for each channel, all channels can be measured within an interval < 1 s. A custom LabView-based software is used to control the instrument, perform calibrations of the sensor spots, and log the measurement data.

#### Calculations

By calculating the difference between the oxygen concentration between the inlet and outlet sensor and multiplying this value by the perfusion velocity, the oxygen consumption of the cells in this chamber can be determined. The oxygen consumption was then normalized to the equilibrium value before adding the substance of interest (baseline).

Oligomycin A, FCCP and rotenone+antimycin A (R/A) were added to obtain insights into mitochondrial respiration. Calculations were performed as described in Divakaruni et al. (2014[14]). Briefly, the following values were determined:

Basal respiration = (last value prior to application of oligomycin A) - (last value after adding R/A)

Proton leak-linked respiration = (last value prior to treatment with FCCP) - (last value after adding R/A)

ATP-linked respiration = (basal respiration) - (proton leak-linked respiration)

Maximal respiration = (last value prior to treatment with R/A) - (last value after adding R/A)

Reserve capacity = (maximal respiration) - (basal respiration).

For the statistical analyses and graphs, Prism 8.4.2 (GraphPad Software Inc., San Diego, CA, United States) was used. Where significance is depicted, a one-way ANOVA with post hoc Tukey test was performed. Substance treatments were performed at least in triplicates (n=3) if not depicted otherwise in the figure caption.

### ATP assay

To analyze the ATP content of cells treated with fructose, an ATP assay was performed (CellTiter-Glo 3D Cell Viability Assay, Promega, Madison, Wisconsin, United States). Non-treated cells were used as negative controls. For treatment and controls, the medium was removed from the inlet and outlet tanks and the inlet tank was filled with 850 µl of either medium supplemented with 40 mM fructose or medium only. After the respective incubation times, the ATP assay was performed using the CyBio FeliX. Briefly, 10 µl of assay reagent was pipetted into the inlet of all cell culture chambers by pressure driven flow. Then, cell lysis was carried out for one hour at room temperature. Afterwards, 99 µl of cell culture medium was pipetted into the outlets by pressure driven flow. Perfused lysate and cell culture medium in the inlet was transferred to a white 96-well plate. The readout was carried out with a Spark multimode reader from Tecan (Männedorf, Switzerland) with 1000 ms integration time.

## Results

**Figure 1:**
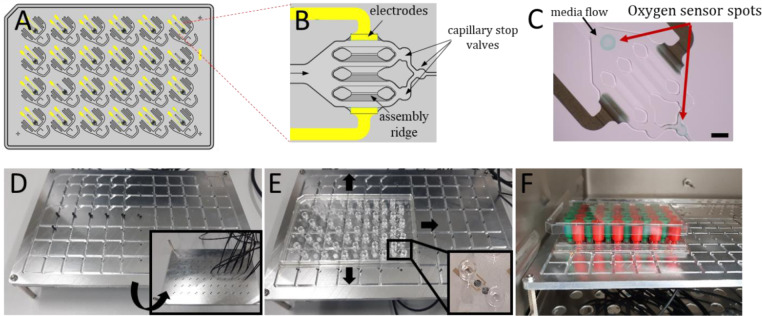
HepaChip-MP with oxygen sensor spots and readout system. A) Schematic overview of the HepaChip-MP with 24 independent cell culture chambers in wellplate format. B) Individual cell culture chamber of the HepaChip-MP showing electrodes (yellow), capillary stop valves to control bubble free filling and three assembly ridges to culture sinusoid-like micro-tissues. Prior to cell assembly the assembly ridges are functionalized by collagen I. C) Microscopic image of cell culture chamber showing the location of the oxygen sensor spots. By measuring oxygen concentration at both the inlet and outlet of the chamber, the oxygen consumption of the microtissue can be determined. D) Photo of the readout system: optical fibers illuminating and probing the oxygen sensors are positioned in holes in the bottom plate to address sensor spots in the HepaChip-MP. E) The HepaChip-MP is positioned by milled guiding lines to precisely align the oxygen sensor spots with the optical fibers. F) The oxygen readout plate is compatible to use in a common incubator. Tanks mounted on the fluidic ports (Luer connectors) of the HepaChip-MP enable continuous perfusion of the cell culture chambers by gravitational driven flow (green: inlet tank; red: outlet tank) (A and B adapted from Busche et al., 2020).

The optical sensor spots located at the inlet and the outlet of the cell culture chamber, respectively, were deposited into the HepaChip-MP using a microdispenser prior to bonding (Figure 1A-C(Fig. 1); Reference in Figure 1: Busche et al., 2020[8]). The resulting sensor coating is shown in Figure 1C(Fig. 1). To align an array of optical fibers with the microplate, an aluminum plate was fabricated with an array of holes to mount optical fibers and tracks to precisely position the HepaChip-MP (Figure 1D, E(Fig. 1)). The HepaChip-MP can be moved on the platform to enable monitoring of the chambers of interest in a parallel fashion. Up to 24 chambers can be measured simultaneously. The platform is compatible with use in common incubators. The HepaChip-MP can easily be retrieved from the platform and handled like common multiwell plates (Figure 1F(Fig. 1)). 

**Figure 2:**
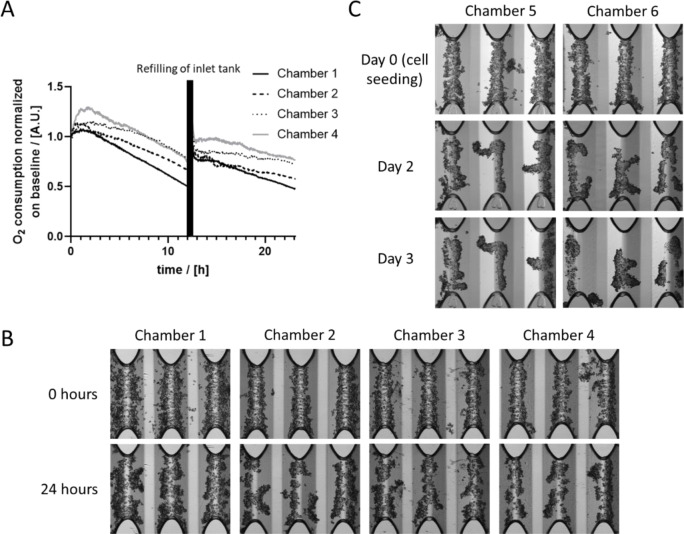
OC of PHH cultured in the HepaChip-MP. A) In cell culture chambers containing microtissues, the OC drops over time. After re-adjusting the medium level difference between inlet and outlet after 12 hours, flow rate increases and the OC immediately rises to a higher level. B) Microscopic images of the cell culture chambers analyzed in A) at the start and the end of the measurement exhibit a slight decrease of cell number. C) Representative images of cells cultured in the HepaChip-MP from the day of seeding (day 0) and day 2 and day 3 of culture. Oxygen measurements after substance treatment were carried out at day 2 and day 3 of the culture in this study.

The OC of PHH was measured for 24 hours in the HepaChip-MP during routine culture. Between medium level adjustments, the OC constantly drops (Figure 2A(Fig. 2)) as the flow rate decreases. After readjustment of medium levels, the OC immediately rises to higher values again. However, OC does not reach the initial level (Figure 2A(Fig. 2)). Microscopy images taken at the start and the end of the experiment showed that the cell number during culture time may slightly decrease during cultivation due to occasional detachment of hepatocytes on the ridges of the culture chambers (Figure 2B(Fig. 2)). Detached hepatocytes may then be flushed out of the chamber. This might explain why the oxygen consumption does not reach the initial levels after readjusting the medium levels. Figure 2C(Fig. 2)) demonstrates that from day 2 to 3 of the culture, the morphology of assembled cell structures does not change much and cell structures seem to be stable. The following experiments to measure oxygen consumption after substance treatment were carried out at day 2 or 3 of culture. Chambers without cells also were analyzed for comparison to rule out oxygen loss through the culture-ware plastic or other non-cellular effects. These chambers show negligible OC (Supplementary Figure 2). 

**Figure 3:**
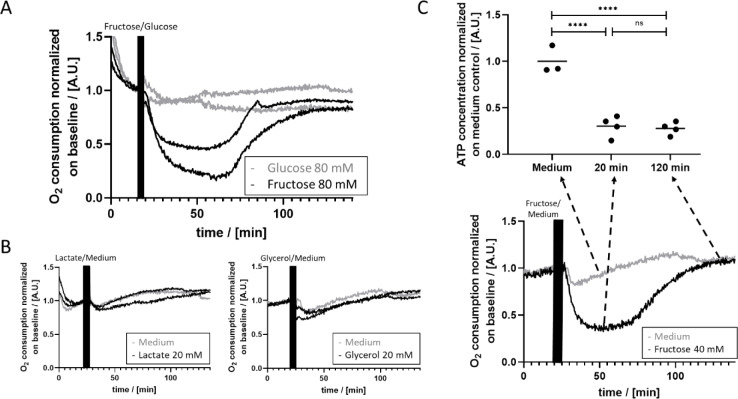
OC and ATP-levels after fructose treatment of PHH. A) While OC rapidly decreases to < 50 % of the basal OC in response to fructose treatment and reaches initial levels again after approximately 110 minutes (black graphs), glucose treatment did not substantially affect OC (grey graphs). B) Cells were treated with 20 mM lactate (left) and 20 mM glycerol (right). Neither lactate nor glycerol affected OC in comparison to control cells treated with medium only. C) The ATP level determined 20 min after the start of the fructose treatment decreased to approximately 30 % of the level of non-treated control cells. Even after reaching close-to-initial levels of cellular OC (illustrated in panel below), the ATP levels did not increase but remained at 30 % of the control cells two hours after the start of the fructose treatment (n = 3 for medium, n = 4 for 20 min and 120 min fructose; significance: **** equals p-values < 0.0001).

Since fructose is well known to interfere with ATP metabolism (Latta et al., 2007[27]), we analyzed the impact of fructose on the OC of PHH. The addition of 80 mM fructose to the medium caused a rapid drop of OC to < 50% of the initial value (Figure 3A(Fig. 3)). Approximately 65 minutes after the onset of fructose treatment, the OC increased again and reached the initial level at 110 minutes. In contrast, incubation with glucose did not cause any change in OC during the 150 minutes of the experiment (Figure 3A(Fig. 3)). Likewise, lactate and glycerol did not show any effect on OC (Figure 3B(Fig. 3)).

Fructose is known to be taken up and phosphorylated by hepatocytes very fast. We tested the ATP concentration of lysed cells after fructose treatment and compared it to cells cultured in standard culture medium. As expected, the ATP concentration after 20 min of fructose treatment was approx. 30 % of the ATP concentration in control cells (Figure 3C(Fig. 3)). Even after apparent equilibration of OC close to the initial level, the ATP concentration of fructose-treated PHH remained low. To test the influence of fructose on mitochondrial respiration of PHH, we analyzed PHH pretreated with 40 mM fructose for 2 and 24 hours. We did not detect any significant differences in cell responses to oligomycin A, FCCP and rotenone+antimycin A compared to cells cultivated in medium only after two hours (data not shown) and 24 hours (Supplementary Figure 3) of fructose treatment. Thus, fructose shows a specific effect on OC right after administration, but does not change mitochondrial respiration after longer treatment.

**Figure 4:**
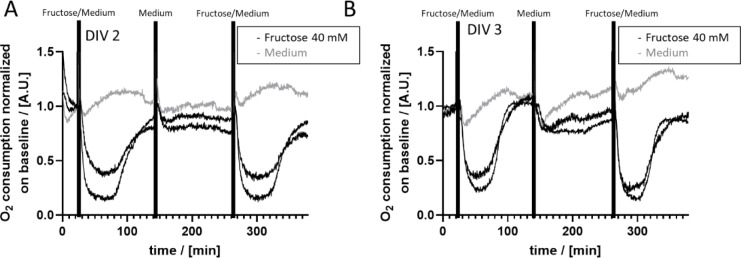
Repeated treatment of hepatocyte cultures by fructose on two consecutive days demonstrates the reproducibility of the effect of fructose on OC. The same cell culture chambers containing PHH were repeatedly treated for 2 hours with 40 mM fructose on DIV (days *in vitro*) 2 (A) and DIV 3 (B). Fructose treatment reproducibly induced the initial decrease in OC followed by an increase to almost initial levels (black graphs). In addition, the intensity of the relative fructose effect on OC is comparable intra-day as well as inter-day. In order to allow the cells to equilibrate to normal metabolic conditions, cells were cultured in standard culture medium for 2 hours in between fructose treatments. Cells cultured in standard medium, did not show substantial changes in OC (grey graphs).

Next, we tested the stability of our system and the cellular regeneration with regard to OC by repeatedly treating PHH cultured in the HepaChip-MP with 40 mM Fructose on DIV (days *in vitro*) 2 and 3, respectively. The response of OC observed was recapitulated after 2 hours of cultivation in medium in between two fructose treatments on DIV 2 as well as on DIV 3 (Figure 4A-B(Fig. 4)). Furthermore, the relative strength of the response to fructose treatment was on similar levels on DIV 2 and DIV 3. 

**Figure 5:**
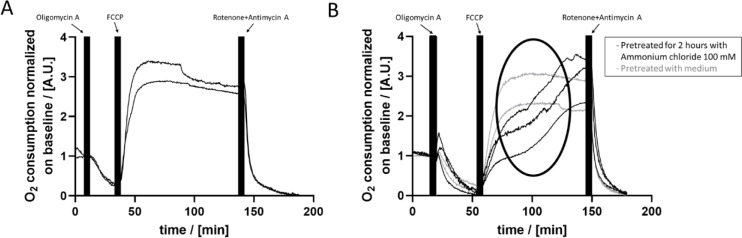
OC of PHH after subsequent application of oligomycin A, FCCP and rotenone+antimycin A (R/A). A) Oligomycin A causes a drop to approximately 25 % of the initial OC. FCCP leads to a rapid increase to > 200 % of the OC, while R/A treatment results in a rapid decrease to negligible OC values. B) Cells were pretreated for 2 hours with 100 mM ammonium chloride (black graphs) or culture medium (grey graphs). Oligomycin A and R/A caused a similar decrease of the OC for pretreated as well as non-pretreated cells as observed in A). However, while the FCCP treatment caused the expected rapid increase until reaching a plateau for non-pretreated cells, cells pretreated with 100 mM ammonium chloride showed a sigmoidal-like increase of the OC thereafter (pointed out by the black ellipse).

Further, we went on to modulate different mechanisms contributing to cellular respiration by adding different substances and recording their impact on OC. First, the addition of oligomycin A resulted in a decrease of OC (Figure 5A(Fig. 5)). Subsequently, incubation with FCCP lead to a rapid increase of OC until reaching a plateau. Finally, after the addition of rotenone+antimycin A (R/A), the OC decreased, reaching a level below the one observed after the addition of oligomycin A. Cells treated with vehicle control (DMSO) did not show any changes in OC (Supplementary Figure 4). Thus, our system shows the expected results on the mentioned substances and can be used to get deeper insights into mitochondrial respiration after substance treatment. As mentioned earlier, fructose for example does initially effect oxygen consumption, but does not induce differences in cell response to oligomycin A, FCCP or R/A treatment (Figure 3(Fig. 3), Supplementary Figure 3).

We tested the influence of ammonium chloride on cellular respiration of PHH. Cells either were pretreated for 2 hours with 100 mM ammonium chloride or medium only (negative control). Oligomycin A treatment did not induce any differences between non-treated and pre-treated cells (Figure 5B(Fig. 5)). Also, the treatment with R/A led to a very similar drop in OC. In contrast, the addition of FCCP yielded differing responses. Pretreated cells showed a sigmoidal-shaped increase of OC while the OC of non-pretreated cells rapidly increased to reach a plateau (Figure 5B(Fig. 5)).

**Figure 6:**
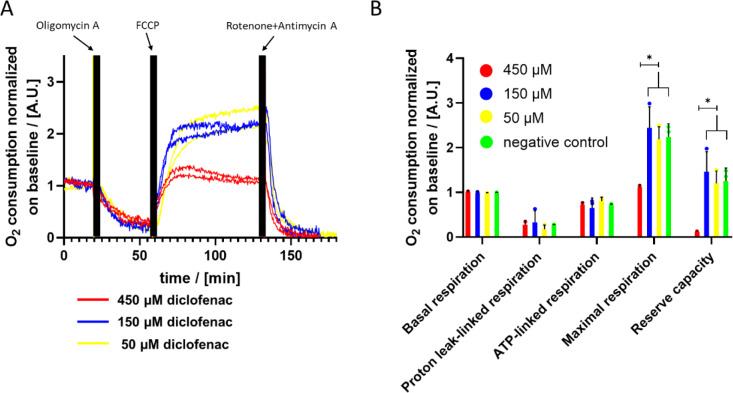
Analysis of the influence of the treatment with different concentrations of diclofenac for 24 hours. A) OC after subsequent application of oligomycin A, FCCP and R/A of PHH pretreated with diclofenac. B) Detailed analysis of cellular respiration of PHH treated with different concentrations of diclofenac (n=2 for 450 µM diclofenac; n=3 for 150 µM and 50 µM diclofenac; significance: * equals p-values < 0.05).

Finally, we tested the influence of diclofenac as a known hepatotoxic drug on the OC of PHH. Cells pretreated with 50 µM, 150 µM and 450 µM of diclofenac showed the expected trend of OC after treatment with oligomycin A, FCCP and R/A (Figure 6A(Fig. 6)). However, the cells pretreated with 450 µM diclofenac reached substantially lower levels of OC after FCCP treatment as compared to cells pretreated with lower diclofenac concentrations (Figure 6A(Fig. 6), solid line). This observation was confirmed by quantification of the maximal respiration and reserve capacity of diclofenac-treated and control cells. PHH pretreated with 450 µM diclofenac show significant lower values for maximal respiration and reserve capacity (Figure 6B(Fig. 6)). For basal respiration, proton-leaked respiration and ATP-linked respiration, no significant differences were observed. PHH pretreated with 1500 µM diclofenac do not show any response in OC on treatment which implicates that they are dead (Supplementary Figure 5) as observed previously (Busche et al., 2020[8]). 

## Discussion

### Novel, parallelized readout system for continuous measurement of OC in an organ-on-chip model

Nutrition plays an important role in liver metabolism and influences the metabolic status of hepatocytes (Jorquera et al., 1996[25]; Walter-Sack and Klotz, 1996[51]). Different diets can also influence drug metabolism (Walter-Sack and Klotz, 1996[51]). Altered drug metabolism on the other hand may lead to drug-induced liver injury (DILI), which is accompanied by mitochondrial disfunction (Corsini and Bortolini, 2013[10]; Pessayre et al., 2010[39]). By measuring the cellular oxygen consumption after substance-challenge, conclusions can be drawn concerning the metabolic status of the cell and the function of mitochondria. Hence, measurement of oxygen consumption is very useful to gain deeper insights into effects of various nutrients and drugs on the liver.

In this work, we introduce a novel platform to analyze the effect of substances on OC and respiration. Optical oxygen sensors were integrated in the previously described HepaChip-MP enabling parallelized readouts (Busche et al., 2020[8]). The sensor platform is compatible for use in common incubators thus enabling continuous monitoring of OC without interference with the cell culture. The HepaChip-MP is developed in the SBS-standard 96-well plate format and can be easily integrated into common cell culture workflows. Unidirectional perfusion results in continuous substance supply at the cells representing a more realistic substance exposure than observed in static cell culture systems for most applications. Cell response to fructose treatment demonstrated stability and robustness as well as intraday and interday reproducibility of OC measurements (Figure 4(Fig. 4)). When compared to the benchmark systems MitoXpress and the seahorse XF analyser (Ferrick et al., 2008[18]; Hynes et al., 2009[24]) the system presented here adds perfusion and thus, a more *in vivo*-like supply of oxygen. In addition, medium and test substances can easily be exchanged during the continuous measurement, which enables numerous consecutive treatments and versatile treatment schemes (see Table 2(Tab. 2); References in Table 2: Ferrick et al., 2008[18]; Hynes et al., 2009[24]; Matsumoto et al., 2018[30]; Moya et al., 2018[32]; Prill et al., 2016[40]; Rennert et al., 2015[42]; Weltin et al., 2017[53]). In contrast to electrochemical oxygen sensors (used by e.g. Moya et al., 2018[32]; Weltin et al., 2017[53]), optical sensors do not consume oxygen. The consumption of oxygen by the sensors would lead to erroneous results in static cultures, in case of very low perfusion rates, low medium volume or under hypoxic conditions. In these conditions, the amount of oxygen is initially already low and by consuming oxygen, the sensors might influence cellular function. Organ-on-chip systems often have low medium volumes and oxygen exchange with the atmosphere may be difficult due to the closed chamber. Hence, optical sensors are preferred in these systems. Optical sensors have already been introduced in organ-on-chip systems (Matsumoto et al., 2018[30]; Prill et al., 2016[40]; Rennert et al., 2015[42]). These systems enable exciting insights in e.g. the establishment of an oxygen gradient (Matsumoto et al., 2018[30]), mechanisms of action of amiodarone (Prill et al., 2016[40]) and the influence of the flow rate on oxygen consumption (Rennert et al., 2015[42]). However, cells are cultured in a 2D configuration which does not resemble the hepatic sinusoid (Matsumoto et al., 2018[30]; Rennert et al., 2015[42]). Or sensor probes are in direct contact to the cells which may influence cell function (Matsumoto et al., 2018[30]; Prill et al., 2016[40]). The HepaChip-MP combines culturing cells in an elongated sinusoid-like pattern under perfusion with non-invasively and continuously measuring the oxygen consumption (Table 2(Tab. 2)).

### Relation of OC and flow rate 

We found that the OC of the cultured PHH decreased over time between readjustments of the difference of medium levels between inlet and outlet reservoirs (Figure 2(Fig. 2)). After readjusting the height difference, the OC is elevated again and starts to decrease until the following refillment. Most probably, this is due to decreasing flow velocities (see Supplementary Figure 1). Supporting this hypothesis, Felder et al. showed that the OC of mesenchymal stem cells decreases with lower flow rates (Felder et al., 2020[17]). Rennert et al. demonstrated that higher perfusion rates lead to a higher total OC in a liver-on-a-chip platform (Rennert et al., 2015[42]). Wei et al. showed that in brains of aged mice both flow rate and OC increase (Wei et al., 2020[52]) suggesting that the relationship between flow rate and OC might also have relevance *in vivo*. It would certainly be interesting to analyze the correlation of flow rate and OC in pathological contexts, e.g. high and low blood pressure or aging.

We also observed that after readjusting the level difference between the inlet and outlet reservoirs, the OC does not reach initial levels anymore but only slightly lower values (Figure 2(Fig. 2)). We attribute this to a small number of cells being flushed out of the culture chamber over time. As a result, fewer cells are present consuming less oxygen. 

Other effects observed upon treatment (e.g. the fructose-effects), however, cannot be attributed to a decrease of cell number. As Figure 4(Fig. 4) shows, the relative intensity of the fructose-induced effect is reproduced well over different days and different chambers while the oxygen consumption of the reference is reproduced over 5-6 hours within a few percent. 

Normalizing OC to cell number would be highly desirable to account adequately for the possible loss of cells. However, chip dimensions, 3-dimensional structure of cell aggregates and perfusion hamper precise cell count in organ-on-chip systems. Therefore, data was normalized to the initial oxygen consumption to determine relative changes in oxygen consumption over the course of an experiment. 

### Fructose effect

Fructose is used extensively as a sweetener for food and especially drinks. Conducted studies have correlated long-term fructose intake with an increased risk of developing liver diseases and diabetes mellitus (for reviews see (Alwahsh and Gebhardt, 2017[2]; Bidwell, 2017[5])). Fructose is also known to be quickly phosphorylated by hepatocytes and thus using a large amount of energy (van den Berghe et al., 1977[48]). Hence, we applied fructose as a model substance in our system to analyze treatment-induced changes of cellular OC. Fructose treatment resulted in an initial drop of OC and a subsequent increase to similar to initial levels within approximately 2 hours after treatment start (Figure 3(Fig. 3)). We used glucose as a control since it consists of the same atoms but has a slightly different structure than fructose. Lactate and glycerol are used as controls because they are products of the fructose metabolism. None of the control substances caused a change in OC of PHH after treatment (Figure 3(Fig. 3)). Therefore, the demonstrated effect of fructose seems to be fructose-specific. Pyruvate also did not show the biphasic effect of fructose on OC after treatment of PHH in preliminary experiments (data not shown). Hence, the availability of NADH does not seem to play an important role in the fructose effect. The biphasic effect of fructose has already been described by Ylikhari et al. in perfused rat livers in 1971 (Ylikahri et al., 1971[54]). To our knowledge, there are not any more recent experiments giving insights into the immediate effect on OC after fructose-treatment of hepatocytes. Ylikhari et al. propose that the initial drop in oxygen consumption is due to depletion of P_i_ and thus inhibition of oxidative phosphorylation (Ylikahri et al., 1971[54]). With increasing concentrations of P_i_, the oxidative phosphorylation is induced again and thus cellular respiration also increases. This would imply that the ATP is regenerated through oxidative phosphorylation. In our experiments however, the ATP concentration stayed low even though the cellular OC increased to the initial level after two hours of fructose incubation (Figure 3(Fig. 3)). This finding suggests that a mechanism different from ATP and P_i_ depletion might be responsible for the change of oxygen usage of PHH after fructose treatment. One might then expect that glycolysis instead of oxidative phosphorylation would fuel ATP recovery in order to regenerate the metabolic equilibrium. However, after fructose treatment ATP level remained low even as oxygen consumption reaches initial levels again. In case of enhanced glycolytic activity, ATP level would be expected to rise independently of oxygen consumption rather than remaining at a low level. Short-term application of fructose is known to protect cells from hypoxia-induced injury and oxidative stress (Lefebvre et al., 1994[28]; Semchyshyn, 2013[44]). Our experiments implicate that 2 hours of fructose-treatment might result in a new equilibrium between cellular OC and ATP concentration (Figure 3(Fig. 3)). The mechanisms behind this equilibrium are still unclear but may give more insight into the protective effect of short-term treatment with fructose.

### Cellular respiration and toxicity measurements

Measuring the uptake of oxygen by cell cultures is an important marker of cell viability and status of cellular metabolism. In general, a higher energy demand of cells leads to higher oxygen uptake while a low energy demand results in lower OC. The consecutive treatment with oligomycin A, FCCP and rotenone+antimycin A (R/A) in combination with a measurement of OC gives insights into cellular respiration and mitochondrial (dis-)function (Divakaruni et al., 2014[14]). Oligomycin A inhibits the ATP synthase and thus the OC decreases after treatment. FCCP functions as an uncoupling agent and rapidly increases OC. Rotenone and antimycin A inhibit the electron transport chain, which leads to the inhibition of OC of mitochondria. Here, we demonstrated that these effects can be reproduced in our system as expected (Figure 5(Fig. 5)). This enables the possibility to analyze the influence of substances on cellular respiration and its mechanisms.

As an example, we treated PHH cultured in the HepaChip-MP with ammonium chloride. Ammonium is known to have toxic effects on different tissues, cells and mitochondria, but the detailed mechanism of action is still unclear (Dasarathy et al., 2017[11]). Niknahad et al. demonstrated mitochondrial toxicity in hepatocytes treating isolated liver mitochondria of mice with ammonia (Niknahad et al., 2017[35]). Mechanisms of action possibly include activation of NMDA receptors, intracellular Ca^2+^ accumulation, opening of mitochondrial permeability transition pores and thus a disturbance of the mitochondrial membrane potential (Bai et al., 2001[4]; Monfort et al., 2002[31]; Rama Rao et al., 2003[41]). We found that PHH treatment with FCCP did not lead to the expected rise in cellular OC if cells are pretreated with ammonium (Figure 5(Fig. 5)). Instead, the OC of pretreated cells rose in a sigmoidal curve in contrast to the fast increase of OC in untreated PHH. This finding was specific for ammonium chloride in our experiments (compare to Figure 6(Fig. 6), Supplementary Figure 3) and might act as a starting point to obtain deeper insights into its toxicity. 

Diclofenac is known to be connected with drug-induced liver injury (DILI) and mitochondrial damage (Boelsterli, 2003[6]). Van Leeuwen et al. reported that diclofenac inhibits cellular respiration by inhibiting subunits of the respiratory chain in *Saccharomyces cerevisiae* (van Leeuwen et al., 2011[49]). We also demonstrated disturbed mitochondrial respiration after treatment with 450 µM diclofenac for 24 hours (Figure 6(Fig. 6)). Syed et al. reported IC_50_ values as low as 19.5 µM for diclofenac in isolated rat mitochondria (Syed et al., 2016[46]). They also demonstrated the protective effect of GSH. Thus, the GSH contained in whole hepatocytes could explain the higher concentration needed to induce toxic effects in our study. In a previous study, we presented diminished viability after diclofenac treatment for 24 hours only at a concentration as high as 1500 µM (Busche et al., 2020[8]). Hence, analyzing the cellular respiration with on-chip oxygen sensors seems to be more sensitive than the resazurine assay, we used in our earlier study to detect diclofenac-induced toxicity. The main differences between cells treated with ammonium chloride or diclofenac and control cells were in the response to FCCP treatment. Zhdanov et al. showed that cell response on FCCP treatment may depend on the nutrient supply and the metabolic status of the cell (Zhdanov et al., 2014[55]). Thus, modulating the cellular microenvironment in combination with FCCP treatment supports in depth analysis of metabolic changes in cells treated with various physiologic and toxic compounds. In summary, the microfluidic system presented here allows extensive investigations of metabolic interactions and DILI by measuring cellular respiration and mitochondrial function.

## Conclusion

We presented an innovative organ-on-chip system to measure oxygen concentration online and non-invasively after substance treatment. This enables the analysis of detailed mitochondrial (dis-)function in pathophysiological situations. The system is parallelized, easy-to-use and integrates well into common cell culture workflows. The demonstration of different effects of fructose, ammonium chloride and diclofenac shows potential to enable analysis of mechanisms of action of diverse substances under continuous and unidirectional perfusion in an advanced *in vitro *model.

## Notes

Marius Busche and Martin Stelzle (NMI Natural and Medical Sciences Institute at the University of Tübingen, Markwiesenstraße 55, 72770 Reutlingen, Germany; Tel.: +49 7121 51530-0, E-mail: martin.stelzle@nmi.de) contributed equally as corresponding author.

## Declaration

### Acknowledgments

We thank Clara Daab, Matthew McDonald and Martin Gaier of the biomedical micro and nano engineering group of the NMI for fabricating the aluminium plate to align the optical fibers with HepaChip-MP.

### Funding

Funding for this research provided by the German ministry for education and research (BMBF) through grant no. 031B0481D is gratefully acknowledged. 

This work received financial support from the State Ministry of Baden-Wuerttemberg for Economic Affairs, Labour and Housing Construction.

### Conflict of interest

M.S. is inventor in patents covering HepaChip-MP technology. J.F. and T.M. are working with PyroScience AT GmbH.

## Supplementary Material

Supplementary information

## Figures and Tables

**Table 1:**
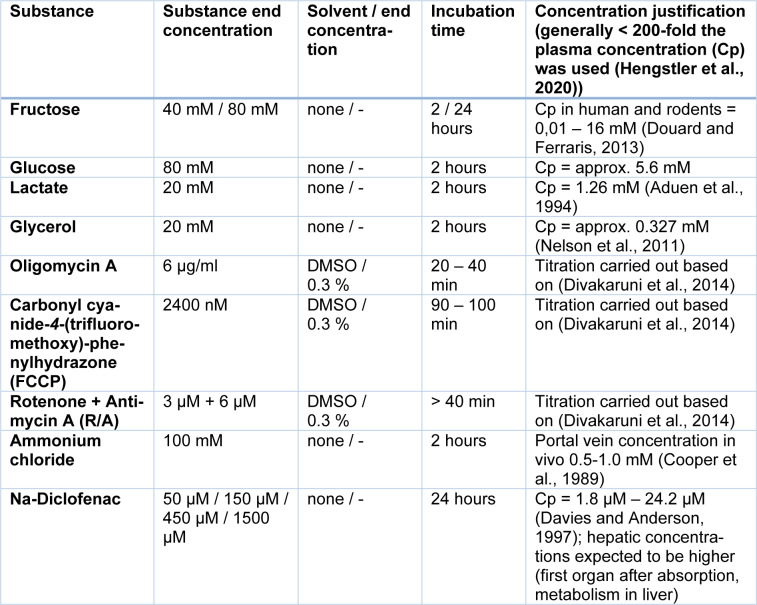
Substances used for treating cells cultured in the HepaChip-MP

**Table 2:**
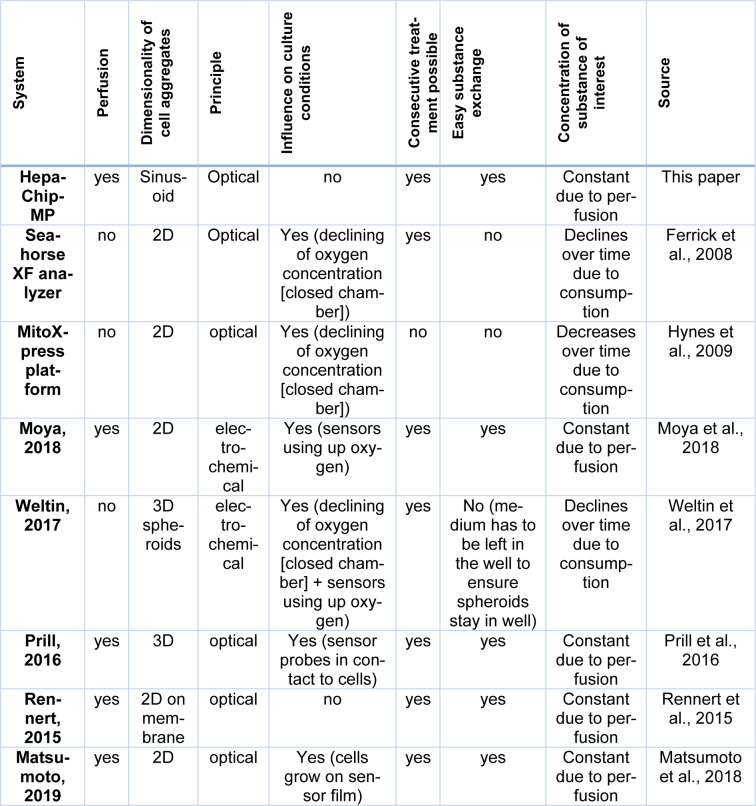
Comparison of features of the HepaChip-MP to other available systems to measure oxygen consumption in cell culture systems
