# Supplementary material for: Continous, non-invasive monitoring of oxygen consumption in a parallelized microfluidic in vitro system provides novel insight into the response to nutrients and drugs of primary human hepatocytes
Source: EXCLI J. 2022 Jan 7;21:144–61. doi: 10.17179/excli2021-4351 (PMC8822303; doi:10.17179/excli2021-4351)
Supplement: Supplementary information [file EXCLI-21-144-s-001.pdf]

EXCLI Journal 2022;21:144-161 – ISSN 1611-2156
Received: September 29, 2021, accepted: November 29, 2021, published: January 07, 2022

Supplementary information to:

Original article:

CONTINUOUS, NON-INVASIVE MONITORING OF OXYGEN CONSUMPTION IN A PARALLELIZED MICROFLUIDIC *IN VITRO* SYSTEM PROVIDES NOVEL INSIGHT INTO THE RESPONSE TO NUTRIENTS AND DRUGS OF PRIMARY HUMAN HEPATOCYTES

Marius Busche[1,*], Dominik Rabl[2], Jan Fischer[3], Christian Schmees[1], Torsten Mayr[2,3], Rolf Gebhardt[4,5], Martin Stelzle[1,*]

[1] NMI Natural and Medical Sciences Institute at the University of Tübingen, Reutlingen, Germany
[2] Institute of Analytical Chemistry and Food Chemistry, Graz University of Technology, Graz, Austria
[3] PyroScience AT GmbH, Aachen, Germany
[4] Rudolf-Schönheimer-Institute of Biochemistry, Leipzig University, Leipzig, Germany
[5] InViSys-Tübingen GbR, Leipzig, Germany

* **Corresponding authors:** Marius Busche and Martin Stelzle, NMI Natural and Medical Sciences Institute at the University of Tübingen, Markwiesenstraße 55, 72770 Reutlingen, Germany, Tel.: +49 7121 51530-0;
E-mails: marius.busche@nmi.de; martin.stelzle@nmi.de

https://dx.doi.org/10.17179/excli2021-4351

This is an Open Access article distributed under the terms of the Creative Commons Attribution License (http://creativecommons.org/licenses/by/4.0/).

EXCLI Journal 2022;21:144-161 – ISSN 1611-2156
Received: September 29, 2021, accepted: November 29, 2021, published: January 07, 2022

1. Perfusion velocity over time in the HepaChip-MP

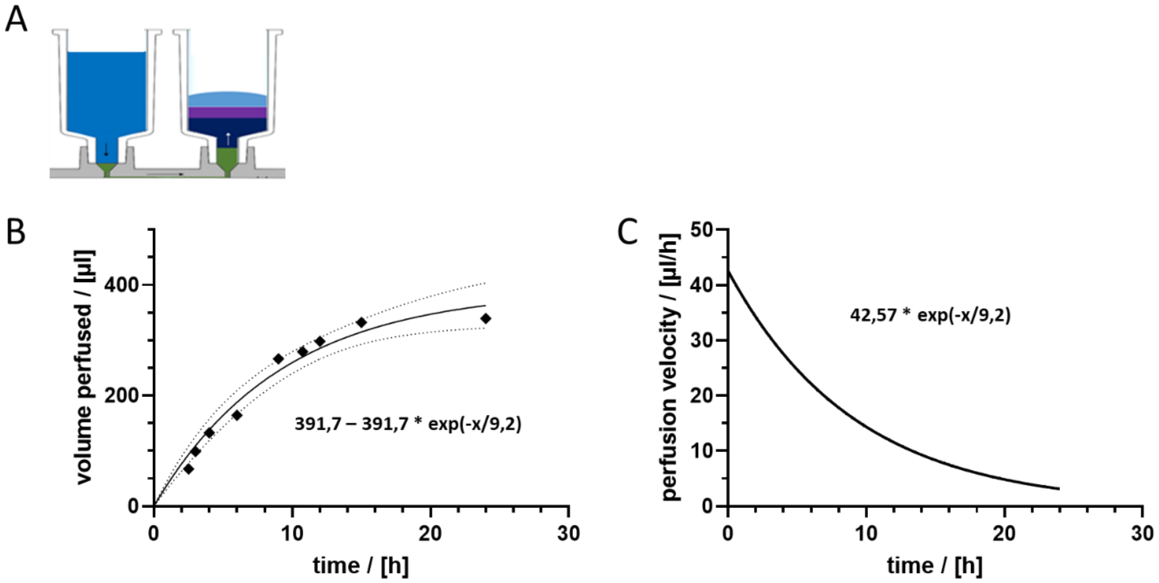

Supplementary Figure 1: Perfusion velocity over time in the HepaChip-MP. **A**) Perfusion is initiated by filling the inlet tank (left) higher than the outlet tank (right). **B**) The perfused volume over time was measured and fitted with the depicted function. By calculating the derivative, the perfusion velocity over time was determined (**C**).

2. Comparison of chambers with assembled cells and empty chambers

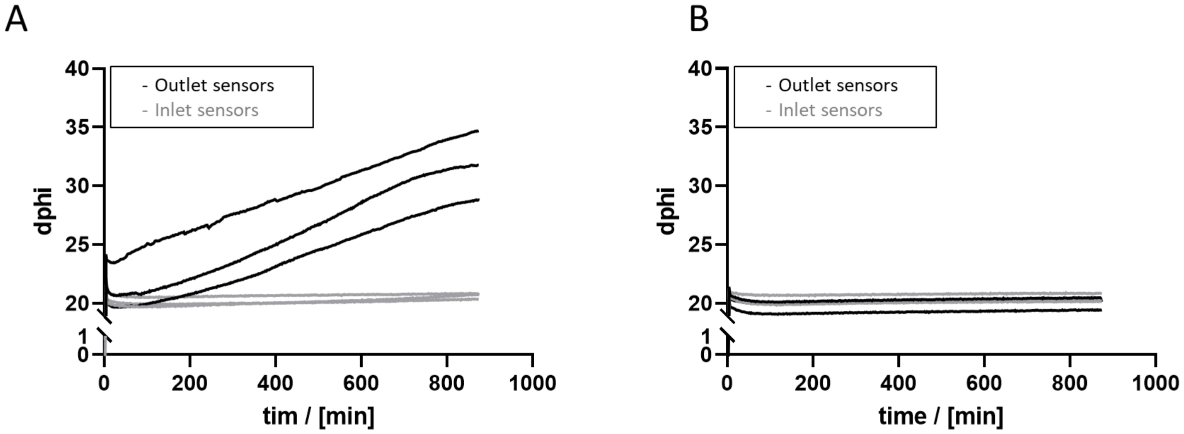

Supplementary Figure 2: Raw data of inlet (grey) and outlet (black) oxygen sensors over 14 hours of routine culture in chambers with assembled PHH (**A**) and without cells (**B**). The oxygen concentration at the inlet of the chambers does not change over time. While the oxygen concentration at the outlet of empty chambers also does not change over time, the oxygen concentration in chambers with assembled cells changes.

3. Long-term effect of fructose

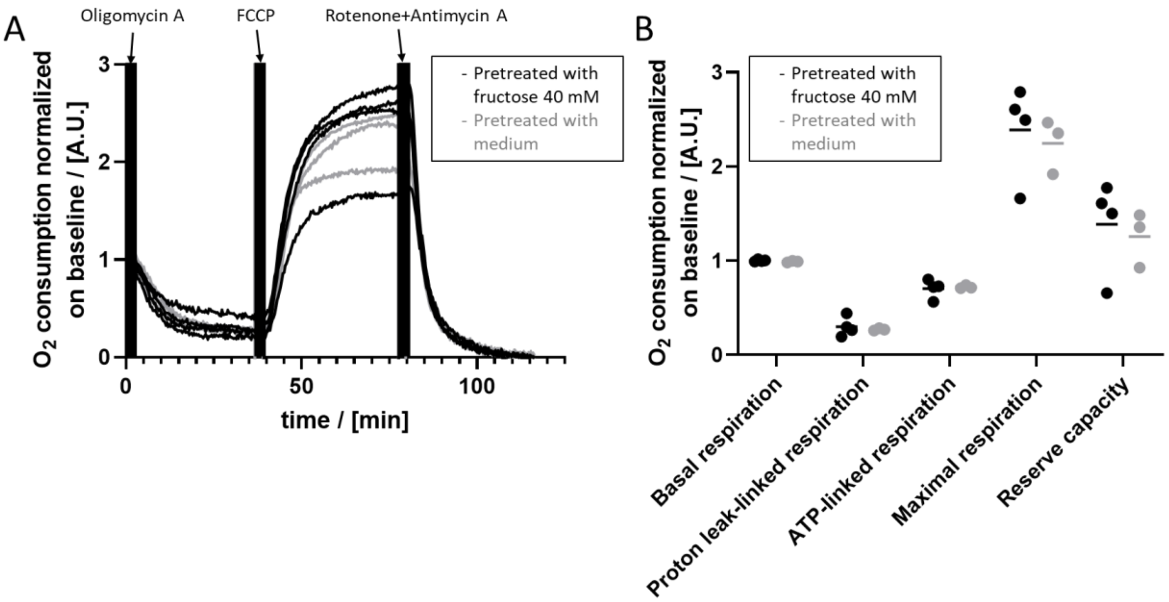

Supplementary Figure 3: Analysis of the influence of the treatment with fructose for 24 hours. **A**) OC of PHH pretreated with 40 mM fructose (black) and medium (grey) after subsequent application of oligomycin A, FCCP and rotenone+antimycin A. **B**) Detailed analysis of cellular respiration of PHH pretreated with 40 mM fructose and medium only. No significant differences could be observed.

4. Mitochondrial respiration and DMSO control

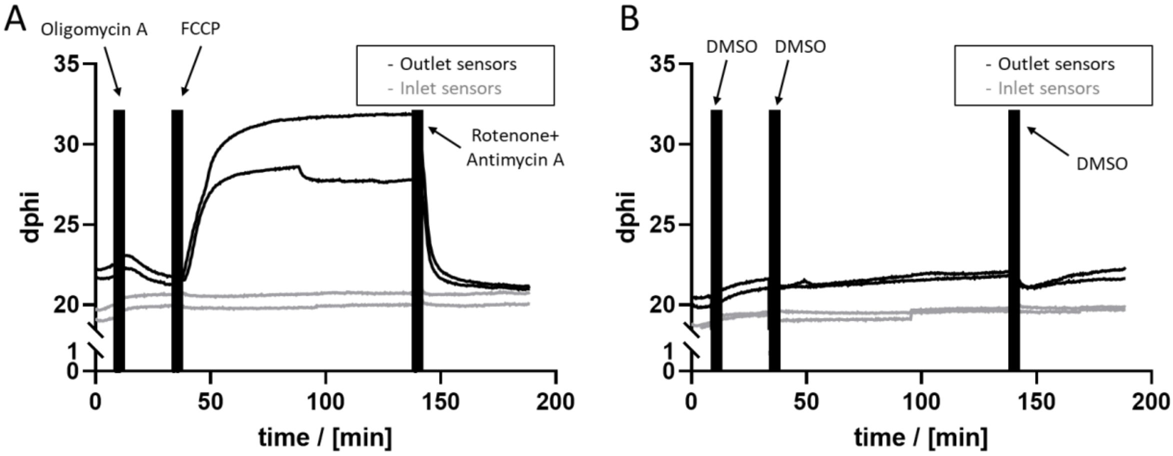

Supplementary Figure 4: Raw data of inlet (grey) and outlet (black) oxygen sensors showing changes after treatment with Oligomycin A, FCCP and rotenone/antimycin A (**A**) and with DMSO as a solvent control (**B**). While the cells treated with the mentioned substances show the expected response, cells treated with DMSO show no effect.

EXCLI Journal 2022;21:144-161 – ISSN 1611-2156
Received: September 29, 2021, accepted: November 29, 2021, published: January 07, 2022

5. 1500 µM of diclofenac kills cells

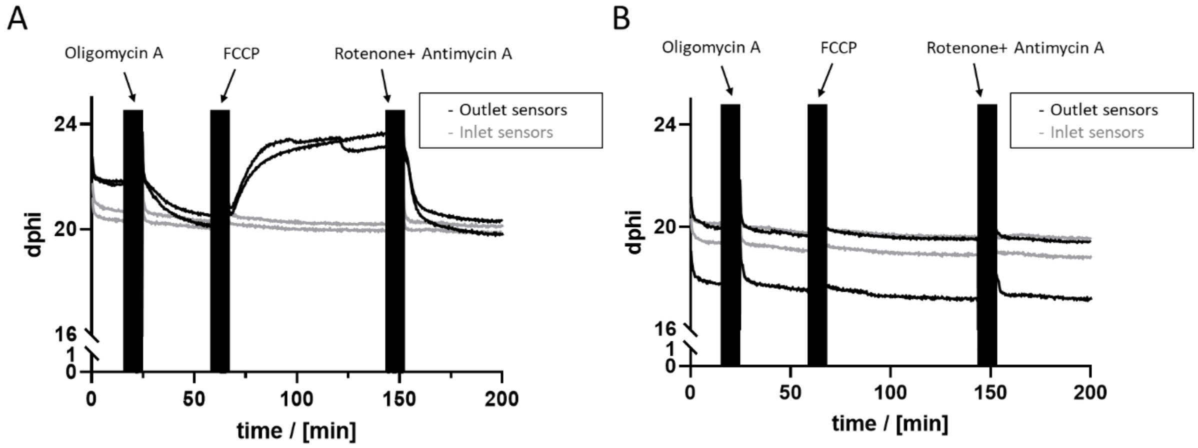

Supplementary Figure 5: Raw data of inlet (grey) and outlet (black) oxygen sensors showing different responses on oligomycin A, FCCP and rotenone+antimycin A treatment of PHH pretreated with 50 µM diclofenac (**A**) and 1500 µM diclofenac (**B**). **A**) PHH pretreated with 50 µM diclofenac show the expected cell response in OC. **B**) PHH pretreated with 1500 µM diclofenac do not show any cell response in OC after substance applications.
